# Methamphetamine-Triggered Neurotoxicity in Human Dorsolateral Prefrontal Cortex

**DOI:** 10.31661/gmj.v10i0.2016

**Published:** 2021-02-02

**Authors:** Ali Zare, Alireza Ghanbari, Mohammad Javad Hoseinpour, Mahdi Eskandarian Boroujeni, Alimohammad Alimohammadi, Mohammad Amin Abdollahifar, Abbas Aliaghaei, Vahid Mansouri, Hamid Zaferani Arani

**Affiliations:** ^1^Young Researchers and Elite Club, Tehran Medical Sciences, Islamic Azad University, Tehran, Iran; ^2^Department of Stem Cells and Regenerative Medicine, Faculty of Medical Biotechnology, National Institute of Genetic Engineering and Biotechnology, Tehran, Iran; ^3^Iranian Legal Medicine Organization, Tehran, Iran; ^4^Biology and Anatomical Sciences Department, School of Medicine, Shahid Beheshti University of Medical Sciences, Tehran, Iran; ^5^Faculty of Paramedical Science, Proteomics Research Center, Shahid Beheshti University of Medical Sciences, Tehran, Iran

**Keywords:** Methamphetamine, Prefrontal Cortex, Apoptosis;, BDNF

## Abstract

**Background:**

Methamphetamine (MA), is an extremely addictive stimulant that adversely affects the central nervous system. Accumulating evidence indicates that molecular mechanisms such as oxidative stress, apoptosis, and autophagy are involved in the toxicity of MA. Considering experimental animal studies exhibiting MA-induced neurotoxicity, the relevance of these findings needs to be evidently elucidated in human MA users. It is generally assumed that multiple chemical substances released in the brain following MA-induced metabolic activation are primary factors underlying damage of neural cells. Hence, this study aimed to investigate the role of autophagy and apoptosis as well as oxidative stress in the brain of postmortem MA-induced toxicity.

**Materials and Methods:**

In this study, we determine the gene expression of autophagy and apoptosis, including BECN1, MAP1ALC3, CASP8, TP53, and BAX genes in ten healthy controls and ten chronic users of MA postmortem dorsolateral prefrontal cortex (DLPFC) by real-time polymerase chain reaction. Also, we applied immunohistochemistry in formalin-fixed and paraffin-embedded human brain samples to analyze brain-derived neurotrophic factor (BDNF). Also, spectrophotometry was performed to measure glutathione (GSH) content.

**Results:**

The expression level of apoptotic and autophagic genes (BECN1, MAP1ALC3, CASP8, TP53, and BAX) were significantly elevated, while GSH content and BDNF showed substantial reductions in DLPFC of chronic MA users. Discussion: Our data showed that MA addiction provokes transduction pathways, namely apoptosis and autophagy, along with oxidative mechanisms in DLPFC. Also, MA induces multiple functional and structural perturbations in the brain, determining its toxicity and possibly contributing to neurotoxicity.

**Discussion:**

Our study showed BDNF-positive cells as well as GSH amount, displayed significant declines in DLPFC of MA user. MA addiction provokes transduction pathways, namely apoptosis and autophagy, along with oxidative mechanisms in DLPFC. However, further investigations are needed to throw light on the cellular and molecular mechanisms that act in the various regions of the addicted brain, especially in DLPFC.

## Introduction

Methamphetamine (MA), is an extremely addictive stimulant that adversely affects the cardiovascular and central nervous systems. Recently, the prevalence of MA use is approximately estimated at 35 million people globally [1,2]. MA can traverse the blood-brain barrier due to its lipophilic property. It disturbs the release and reuptake of neurotransmitters, which causes ecstasy, excitement, and attentiveness [3]. Moreover, it could cross membranes independent of dopamine, norepinephrine, and serotonin transporters at high doses, leading to brain damage [4]. Previous reports in animals have shown that high doses of MA could be detrimental for dopamine neurons [5,6]. The clinical manifestation of MA addiction includes insomnia, aggression, psychosis, and physiological signs (such as elevated blood pressure and body temperature) [7]. Further, brain studies in human abusers revealed long-lasting structural changes in white matter [8]. Additionally, a recent study disclosed a raised risk for developing Parkinson's disease in hospitalized MA abusers [9]. The dorsolateral prefrontal cortex (DLPFC) lies in the prefrontal cortex of the brain. This area plays vital role in working memory, language processing, and cognitive control of behavior [10,11]. Moreover, it has been proposed that DLPFC precludes risky decision making (i.e., drug cravings), but this function is interrupted in substance-dependent individuals [12]. Structural imaging findings have also demonstrated a significant reduction of grey matter density in DLPFC of addicted subjects and poor executive function [13]. Accumulating evidence indicates that molecular mechanisms such as oxidative stress and apoptosis are involved in the toxic effects of MA [14]. Furthermore, a growing body of studies emphasizes autophagy, as an evolutionarily conserved mechanism for energy homeostasis, in MA-induced cell death [15]. Also, recent findings reveal the mutual interaction between the brain-derived neurotrophic factor (BDNF) and oxidative stress. For instance, in an animal study, enhanced oxidative stress led to a decrease in BDNF expression, which was reversed by vitamin E supplementation [16]. Considering experimental animal studies exhibiting MA-induced neurotoxicity, these findings' relevance needs to be evidently elucidated in human MA users. Thus, the present study was performed to assess the gene expression of generally accepted markers of autophagy and apoptosis as well as the level of BDNF protein and glutathione (GSH) content in postmortem DLPC of MA abusers.

## Materials and Methods

### 1. Subjects and Brain Sample

A standardized protocol was employed to collect postmortem brain tissues from ten controls and ten chronic MA users in the legal medicine center of Kahrizak, Iran. The DLPC autopsy and immunohistological analysis were performed by fixing one half-brain in formaldehyde fixative. The other half associated with the DLPC was kept in an RNA stabilization solution immediately after the autopsy and frozen for real-time polymerase chain reaction (PCR). Drug screening was also performed by collecting the blood samples of the MA users and controls. All MA users met the following selection criteria: (a) presence of MA on toxicology screens in blood and autopsied brain (b) evidence from the case records and structured interviews with the medical examiner, next of kin, and informants, of use of MA as the primary drug of abuse for at least one year prior to death; and (c) absence of neurological illness from case records or brain pathology unrelated to use of the drug. No neurological disorders were observed in the controls, and no evidence of brain pathologies was identified in their neuropathological examinations. With no history of drug abuse, and blood tests and brain autopsy were also negative for drug use; hence, traumas and/or cardiovascular diseases were their cause of death.

### 2. Immunohistochemistry

Phosphate buffered saline (Sigma Aldrich, USA), containing 1% bovine serum albumin (BSA; Sigma Aldrich, USA) and 0.3% Triton X 100 (Sigma Aldrich) was employed for diluting the primary antibody in which the sections were incubated against the BDNF (1:500) overnight at 4°C using the substrate of the avidin-biotin complex (ABCAM, UK). Afterward, they were treated in 0.05M Tris-buffer (PH=7.6, Sigma Aldrich, USA) with 0.05% 3,3'-Diaminobenzidine tetrahydrochloride and 0.03% hydrogen peroxide (ABCAM, UK), counterstained and mounted after immunohistochemical reactions and ultimately observed and assessed under a light microscope (Nikon, Japan).

### 3. Measuring GSH Levels

The colored product of 2-Nitro-5-thiobenzoic acid (Sigma Aldrich, USA) produced by reductions in GSH caused by 5,5′-dithiobis (2-nitrobenzoic acid) (DTNB, Sigma Aldrich, USA) was quantified at 412 nm. Furthermore, the tissue lysis buffer was employed to lyse the brain tissues, and the Bradford test was conducted to evaluate protein concentrations in the specimens. A 50 μl of the DTNB solution, 0.84 ml of distilled water, and 0.1 ml of a Tris solution were added to prepare the working reagent of DTNB. After carefully mixing this solution, it underwent spectrophotometry (Eppendorf). Also, 10 μl of the tissue lysis buffer was then added to 0.99 ml of the DTNB reagent, stirred well, and incubated at ambient temperature for five minutes. Furthermore, GSH concentrations were expressed as mmol/g of protein.

### Sperm Analysis

In order to analyze sperm, four attributes, including morphology, viability, concentration, viability, and motility of sperm were evaluated.So we first collected the semen from the epididymal tissue. Sperm (10 μL) were transferred to a hemocytometer (Z359629, Merck, Germany), and sperm counts were performed under an optical microscope (BM180N, Novel, China) with a magnification of 40X.Sperm motility was evaluated by a microscope in ten fields based the World Health Organization recommendation.Sperm 3-(4, 5-dimethylthiazol-2-yl)-2, 5-diphenyltetrazolium bromide (MTT; Sigma, USA) viability assay introduced by Nasr-Esfahani (2002) was used to evaluate sperm viability [19].Alanine blue staining (Sigma, USA)was also used to study sperm morphology. The slides were evaluated for morphological disorders in the tail, neck or head.

### 4. PCR

4.1. Extracting RNA and cDNA synthesize High pure RNA tissue kits were employed to extract total RNA as per the manufacturer's protocol (Roche, Switzerland). The reverse transcriptase of Moloney murine leukemia virus (Fermentas, Lithuania) was then used in the presence of RNase inhibitors and random hexamers for the transcription of 1 μg of total RNA into cDNA. 4.2. Quantitative Real-Time PCR According to Table-1, specific primers for MAP1ALC3, BECN1, CASP8, BAX, and TP53 genes were employed to perform quantitative real-time PCR considering GAPDH the internal control. Reactions were conducted by utilizing SYBR® Premix Ex Taq II (Takara, Japan) and a RotorGene™ 6000 machine (Qiagen, Germany).Moreover, initial denaturation was carried out for 15 minutes at 95°C followed by 40 denaturation cycles at the same temperature for five seconds in primer-specific conditions and extension for 20 seconds at 60°C. The candidate groups were compared in terms of the results of quantitative PCR in REST-2009 (Qiagen, Germany).

### 5. Ethical Issues

This research was approved by the Research Ethics Committee associated with Shahid Beheshti University of Medical Sciences (IR.SBMU.REC.1399.104).

### 6. Data Analysis

The data expressed as mean±SEM and were analyzed by SPSS version 23 SPSS Inc., Chicago, IL, USA) using the independent t-test. A P<0.05 was set as the level of statistical significance.

## Results

**Figure 1:**
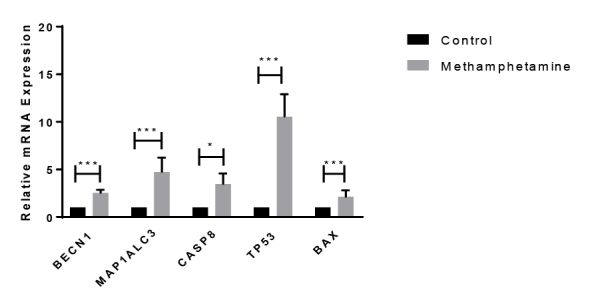
Quantitative real-time PCR for the selected autophagy and apoptosis genes revealed MA-related gene expression changes. The up-regulation of autophagy genes (BECN1 and MAP1ALC3), along with increases in expression levels of apoptotic genes (CASP8, TP53, and BAX). *P<0.05 and *** P<0.001 vs. control group.

**Figure 2:**
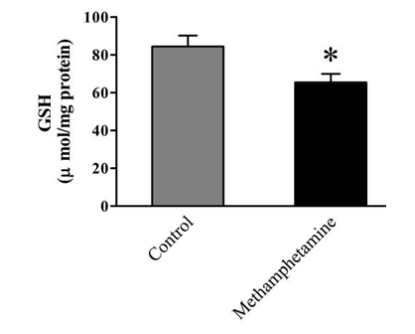
The level of GSH showed a significant decline in DLPC of the MA group compared to the control. *P<0.05 vs. control group.

**Figure 3:**
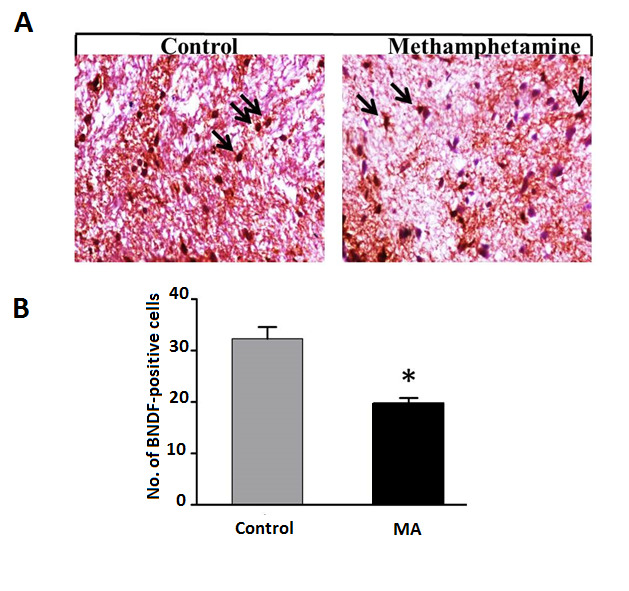
Immunohistochemical staining of BDNF in DLPFC of control and MA group (case No. 2 and 15, respectively).** A: **Arrows indicate BDNF-positive cells (original magnification 20×).** B: **The mean number of BDNF-positive cells decreased significantly in MA users compared to the control subjects. *P<0.01 vs. control group.

Patients Characteristics Patients' characteristics were summarized in Table-2. Autophagy and Apoptosis Markers Change in DLPC of MA Users Quantitative real-time PCR for the selected autophagy and apoptosis markers showed considerable expression changes in the DLPFC of MA users (Figure-1). The up-regulation of autophagy markers BECN1 and MAP1ALC3 were detected in the DLPFC of the MA group (P<0.001). Likewise, the expression levels of specific apoptotic genes such as CASP8, TP53, and BAX showed an increase (P<0.05). MA Exposure Decreases GSH Content in DLPC As shown in Figure-2, the level of GSH demonstrated a nearly 1.4-fold decrease in DLPC of the MA group compared to the control (P<0.05).BDNF-Positive Cells Show a Reduction in MA Group The postmortem brain sections of control and MA groups were immunohistochemically stained for BDNF (Figure-3A). According to our results, the mean number of BDNF-positive cells declined significantly in MA users compared to the control (P < 0.01, Figure-3B).

## Discussion

In this study, the expression level of apoptotic and autophagic genes (BECN1, MAP1ALC3, CASP8, tP53, and BAX) were significantly elevated, while GSH content and BDNF showed substantial reductions in DLPFC of chronic MA users. Acute MA exposure provoked long-lasting damage to monoaminergic nerve terminals in the striatum, hippocampus, and prefrontal cortex, leading to a decline in dopamine release [17]. This deficit might adversely affect the executive function of DLPFC in the modulation of intentional deeds [18]. Moreover, significant reductions in dopamine transports were also reported, particularly in DLPFC, which probably explained MA-related psychiatric symptoms in MA subjects [19]. Autophagy is a mechanism of high importance to preserve cellular homeostasis. Recently, considerable attention has been paid to MA-induced autophagy. For instance, Larsen et al. [20] initially showed the formation of autophagic granules in dopaminergic neurons upon exposure to MA. This finding was also confirmed in similar studies by Fornai et al. [21] and Kanthasamy et al. [22]. Similarly, ubiquitin-positive structures were detected in the midbrain neurons of MA users, suggesting the link between autophagy and MA neurotoxicity [23]. Based on numerous research studies, MA causes neuronal apoptosis through activation of extrinsic /intrinsic death pathways mediated by up-regulation of the pro-apoptotic genes like BAX, BAK, and BAD while down-regulation of anti-apoptotic genes [24]. Besides, it is well-established that the transcription factor, P53 serves a key role in MA-induced cell death through promoting apoptotic pathways [25]. Accordingly, our gene expression data of apoptotic and autophagic genes are in good agreement with past reports [22]. BDNF, as a member of the neurotrophin family, serves important roles in neuronal differentiation and maintenance, neuroprotection, and synaptic plasticity [26]. Dysregulation of BDNF and its main receptor, TrkB, has been documented in neurodegenerative disorders such as Parkinson's disease, Alzheimer's disease, and psychiatric diseases [27]. For example, decreased BDNF production at mRNA and protein levels within the DLPFC of patients with schizophrenia was shown by Weickert et al. [28]. Likewise, decreases in BDNF transcripts were detected in the cortico-hippocampal circuit of people with schizophrenia, highlighting the disturbance of synaptic plasticity-related signaling pathways BDNF-TrkB signaling [29]. The impact of MA regarding disturbing the balance between reactive oxygen species (ROS) generation and the capacity of the antioxidant system to remove ROS has been widely investigated [30]. Excessive release of dopamine triggered by MA has been associated with increased levels of oxygen-based free radicals and dopamine quinones followed by reductions in GSH content [31,32]. Correspondingly, upon MA treatment, augmented oxidative stress is linked with decreases in GSH peroxidase enzymatic activity in differentiated SH-SY5Y neuronal cells [33]. Exposure to MA promotes differential gene expressions leading to changes in levels of inﬂammatory cytokines, neurotrophins (chiefly BDNF) along with the alteration of oxidative stress-linked cascades and apoptotic events [34,35]. One of the most important limitations of the current study was access to a sufficient number of brain samples due to the need to satisfy the families of the deceased.

## Conclusion

Our study showed BDNF-positive cells as well as GSH amount, displayed significant declines in DLPFC of MA user. MA addiction provokes transduction pathways, namely apoptosis and autophagy, along with oxidative mechanisms in DLPFC. However, further investigations are needed to throw light on the cellular and molecular mechanisms that act in the various regions of the addicted brain, especially in DLPFC.

## Acknowledgment

None.

## Conflict of Interest

The authors declare that they have no competing interests.

**Table 1:** Primer Sequences for Real-Time PCR

**Primers**	**Sequence**	**Annealing**	**Accession**
**GAPDH**	F GTCAGTGGTGGACCTGACCT	60ºC × 25 sec	NM_002046.6
	R CACCACCCTGTTGCTGTAGC		
**BECN1**	F GGCTGAGAGACTGGATCAGG	60ºC × 25 sec	NM_003766.4
	R CTGCGTCTGGGCATAACG		
**MAP1ALC3**	F GAGAAGCAGCTTCCTGTTCTGG	60ºC × 25 sec	NM_022818.4
	R GTGTCCGTTCACCAACAGGAAG		
**CASP8**	F ATTAGGGACAGGAATGGAACAC	60ºC × 25 sec	NM_001228.4
	R GGAGAGGATACAGCAGATGAAG		
**TP53**	F GAGGTTGGCTCTGACTGTACC	60ºC × 25 sec	NM_000546.5
	R TCCGTCCCAGTAGATTACCAC		
**BAX**	F TGCCTCAGGATGCGTCCACCAA	60ºC × 25 sec	NM_138761.3
	R CCCCAGTTGAAGTTGCCGTCAG		

**Table 2:** Characteristics of Control and MA Users in this Study.

Case No.*	**Sex**	**Age (years)**	**Postmortem interval (h)**
1	M	36	6
2	M	40	7
3	M	32	11
4	M	29	13
5	M	45	8
6	M	43	5
7	M	34	7
8	M	37	15
9	M	41	19
10	M	39	5
11	M	56	4
12	M	47	9
13	M	28	7
14	M	33	5
15	M	41	11
16	M	27	7
17	M	26	19
18	M	49	21
19	M	40	18
20	M	44	8

**M: **Male *Blood MA levels: 1-19 mg/ml (cases 11-20), negative (cases 1-10)
